# Anchor-Based Whole Genome Phylogeny (ABWGP): A Tool for Inferring Evolutionary Relationship among Closely Related Microorganims

**DOI:** 10.1371/journal.pone.0014159

**Published:** 2010-11-30

**Authors:** Anchal Vishnoi, Rahul Roy, Hanumanthappa K. Prasad, Alok Bhattacharya

**Affiliations:** 1 School of Information Technology, Center for Computational Biology and Bioinformatics, Jawaharlal Nehru University, New Delhi, India; 2 Indian Statistical Institute, New Delhi, India; 3 Department of Biotechnology, All India Institute of Medical Sciences (AIIMS), New Delhi, India; 4 School of Life Sciences, Jawaharlal Nehru University, New Delhi, India; American Museum of Natural History, United States of America

## Abstract

Phenotypic behavior of a group of organisms can be studied using a range of molecular evolutionary tools that help to determine evolutionary relationships. Traditionally a gene or a set of gene sequences was used for generating phylogenetic trees. Incomplete evolutionary information in few selected genes causes problems in phylogenetic tree construction. Whole genomes are used as remedy. Now, the task is to identify the suitable parameters to extract the hidden information from whole genome sequences that truly represent evolutionary information. In this study we explored a random anchor (a stretch of 100 nucleotides) based approach (ABWGP) for finding distance between any two genomes, and used the distance estimates to compute evolutionary trees. A number of strains and species of Mycobacteria were used for this study. Anchor-derived parameters, such as cumulative normalized score, anchor order and indels were computed in a pair-wise manner, and the scores were used to compute distance/phylogenetic trees. The strength of branching was determined by bootstrap analysis. The terminal branches are clearly discernable using the distance estimates described here. In general, different measures gave similar trees except the trees based on indels. Overall the tree topology reflected the known biology of the organisms. This was also true for different strains of *Escherichia coli*. A new whole genome-based approach has been described here for studying evolutionary relationships among bacterial strains and species.

## Introduction

Current understanding of phylogenetic relationship among different organisms is essentially based on rRNA sequences. A number of other genes or a group of genes have also been used for construction of phylogenetic trees [1], [2]. Though a number of predictions match our biological understanding there are problems associated with such approaches (for discussion see Henz *et al* [3]). For one, these approaches do not resolve terminal branches inherent in a group of closely related organisms, such as strains of a species [4]. Occasionally different regions of genomes evolve differently and approaches based on single or a small set of genes may not capture the evolutionary history of these organisms [5].

Whole genome sequences were used instead and approaches based on it can be broadly classified into three categories essentially based on, 1) sequence alignment [4], [6], 2) information content in the form of gene content or gene order [7]–[10] and 3) sequence statistics, such as occurrence of k-mers [11]. Alignment-based methods have been in use ever since Woese first demonstrated rRNA sequence based phylogenetic trees [12]. The accuracy of these methods depend on correct alignment. The accuracy of alignment decrease with its length due to large number of possibilities [13]. Moreover, alignment-based methods do not capture other evolutionary processes, such as insertions and deletions.

Alignment based methods are difficult to apply at the whole genome level due to the problem of alignment. The gene content of genomes can vary due to forces, such as loss and duplication of genes. These may lead to discrepancies in phylogeny in both closely and distantly related genomes [14], for example, they fails to give a correct relationship when closely related genomes share less number of genes because of secondary loss due to adaptation in different ecological niche or due to duplication of genes [15]. In the latter situation the genome distance can be computed by using duplicated genes to estimate the additive genome distance [16]. Also, different homology cutoff is used to remove the discrepancies in gene content tree [17]. Gene order has also been used for estimation of phylogenetic relationship of closely related genomes [18]. However, trees based on gene order, lack resolution as there are very few genome rearrangements observed in nature [19]. In general gene order has low resolving power and gene content may not always reflect true evolutionary history [20], [21]. A tree of life has been constructed using maintenance of protein domain order at the whole genome level as a distance parameter [22]. Algorithm such as MAUVE is used to circumvent many of problem discussed here [23]. But, this analysis can not be extended to the study of closely related organisms due to the problem of sorting out terminal branches. Insertions and deletions in various proteins are also used for construction of phylogeny [24]. Single nucleotide indels has also been used in similar studies [25]. Gene networks, concatenation of genes are also used for the reconstruction of the phylogeny [26], [27], [28].

There are alignment free approaches, such as those based on k-string [11]. The alignment free methods can not be used to understand biological basis of evolution as these have a major problem of not considering evolutionary mechanisms for construction of genome trees. Their major advantage is being computationally less expensive and using maximum content of the genomes.

It is clear from the above discussion that genome trees derived using different parameters can circumvent some of the problems caused by the use of a single measure. The results obtained by Wolf *et al* using five different approaches for the construction of phylogeny show that it is also important to formulate proper methods for computing genomic distance in order to get biologically meaningful trees as there is incongruence in trees generated [19]. Rokas *et al* showed that improved genome wide sampling of unrelated genes can circumvent some of the problems [29].

In this report we describe a method, named anchor-based whole genome phylogeny or ABWGP for determining the phylogenetic relationship based on whole genome sequences without using large scale alignment. The method has been applied to two groups of organisms, closely related species and strains of *Mycobacterium tuberculosis* and different strains of *Escherichia coli*. It is based on the identification of random anchors and their homologs in a pair of genomes as described before [30]. Our approach is different from the several gene used in construction of phylogeny, instead we used small snippets of genome named anchors [30]. These are processed in terms of sequence divergence, inter-anchor distances and anchor order in order to determine pair wise inter-genomic distances. Distance based phylogenetic trees were then constructed using each parameter. An attempt was also made to construct a unified multi-parameter-based tree to understand the true evolutionary relationships. The results were analyzed keeping in view known biology of the organisms.

## Results

### Random sampling and Score Calculation

The approach used in this study is based on random sampling as described earlier [30]. Briefly, a number of sequences of 100 contiguous nucleotides were extracted from random locations of the query genome S. These sequences are referred to as anchors. The BLAST algorithm was used to find the homologs of each anchor in the target genome T. The mismatch score for each anchor was recorded and a normalized score was computed as described in Methods (Fig S1). These were converted into cumulative normalized scores (CNS) utilizing the data from all the random samples. CNS was computed for all pairs of genomes under study. The positions of all homologous anchors in a pair of genomes can be processed to determine incidences of duplication, insertion and recombination as described before [30]. The changes were then converted into distance measures as elaborated in Methods for generating trees.

The length of 100 was chosen for defining anchors due to low probability of finding by chance a match for this length of sequence in a genome. This can be shown as follows. The match of an anchor in a genome has binomial distribution. Due to large sizes of genomes, this can be approximated by a Poisson distribution. If the size of genome is 4,500,000 (generally the size of a Mycobacterial genome), the probability of finding a fixed given sequence of length 100 in a genome of this size is less than 2.8 * 10^−53^ by a simple Poisson approximation of a binomial.

### Minimal Amount of Data needed for Phylogenetic Tree Construction

**Figure 1:**
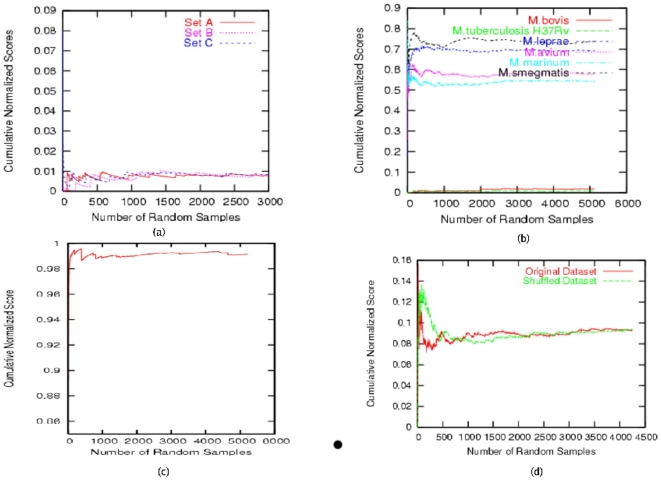
The distribution of Cumulative Normalized Score. The CNS distribution of when the random anchors of (a) *M. tuberculosis* H37Rv (S) with *M. tuberculosis* CDC1551 (T) in three different set of experiment. The CNS converged to similar values with more than 3000 anchors. (b) *M. tuberculosis* CDC1551(S) when was compared with *M. tuberculosis* H37Rv, *M. bovis*, *M. leprae*, *M. avium*, *M. ulceran*, *M. gilvum* (T). Different values of CNS depict phylogenetic distances of *M. tuberculosis* CDC1551(S) with other genomes. (c) CNS distribution when *M. tuberculosis* CDC1551 compared with random genome with the same base composition. (d) The distribution of Cumulative Normalized Score (CNS) when the random anchors were shuffled.

It has been pointed out earlier that CNS was computed from each individual mismatch score of anchors. From Kolmogorov's law of large numbers it can be shown that under very mild assumptions on the structure of a genomic sequence, CNS would attain a stable level when the number of anchors involved in the computation of CNS is large. As can be seen from Fig. 1a the value of CNS reached a steady state after about 3000 anchors. CNS for two closely related genomes was around zero (Fig. 1b) whereas the value was around 0.85 when the genomes are highly divergent, such as a randomly generated sequence and the genome of *M. tuberculosis* (Fig. 1c). The distance measure obtained from CNS was clearly a “random” distance in the sense that two distinct random samples may not yield the same CNS and so the distance depends on the sample chosen. However, as shown in Fig. 1a the CNS is quite “stable” vis-a-vis different random samples, in the sense that there is not a significant difference between the value of CNS obtained from two distinct random sequences (except may be in pathological situations as discussed later). The values of CNS using *M. tuberculosis* CDC1551 (S genome) and other Mycobacterial species (T genome) are shown in Fig. 1b. The anchor samples were also shuffled to see if there was any association between the random samples. There was no such association as both the plots, one for the original data set and another for the shuffled data set converged to the same CNS (0.081) (Fig 1d).

### Properties of Distance Measure

The distance we obtained was also not a metric, i.e. the triangular inequality D(S,T) = D(T,R)> = D(S,R) need not hold. Although D(S,S) = 0, it could be that D(S,T) = 0 for two distinct sequence S and T. However the violation of these properties, rather than being the norm, are generally in exceptional cases like artificially constructed sequences as described later.

The construction of D(S,T) ensures that

- D(S,T)> = 0 and
- D(S,T) = D(T,S)

The latter being obtained because of the symmetrization involved in the construction of D(S,T).

### Construction of Phylogenetic Tree

#### CNS-based tree

**Figure 2:**
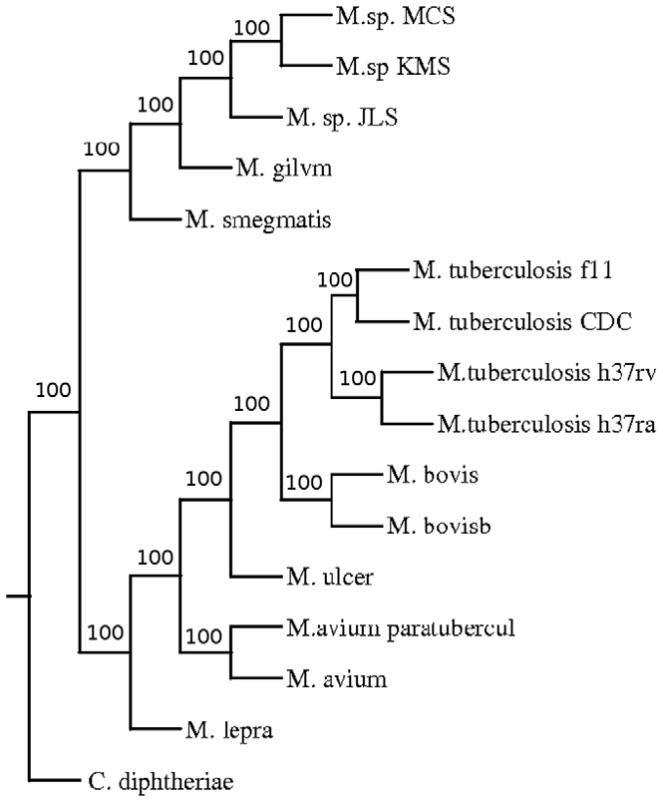
Phylogenetic tree of Mycobacterium based on CNS.

**Figure 3:**
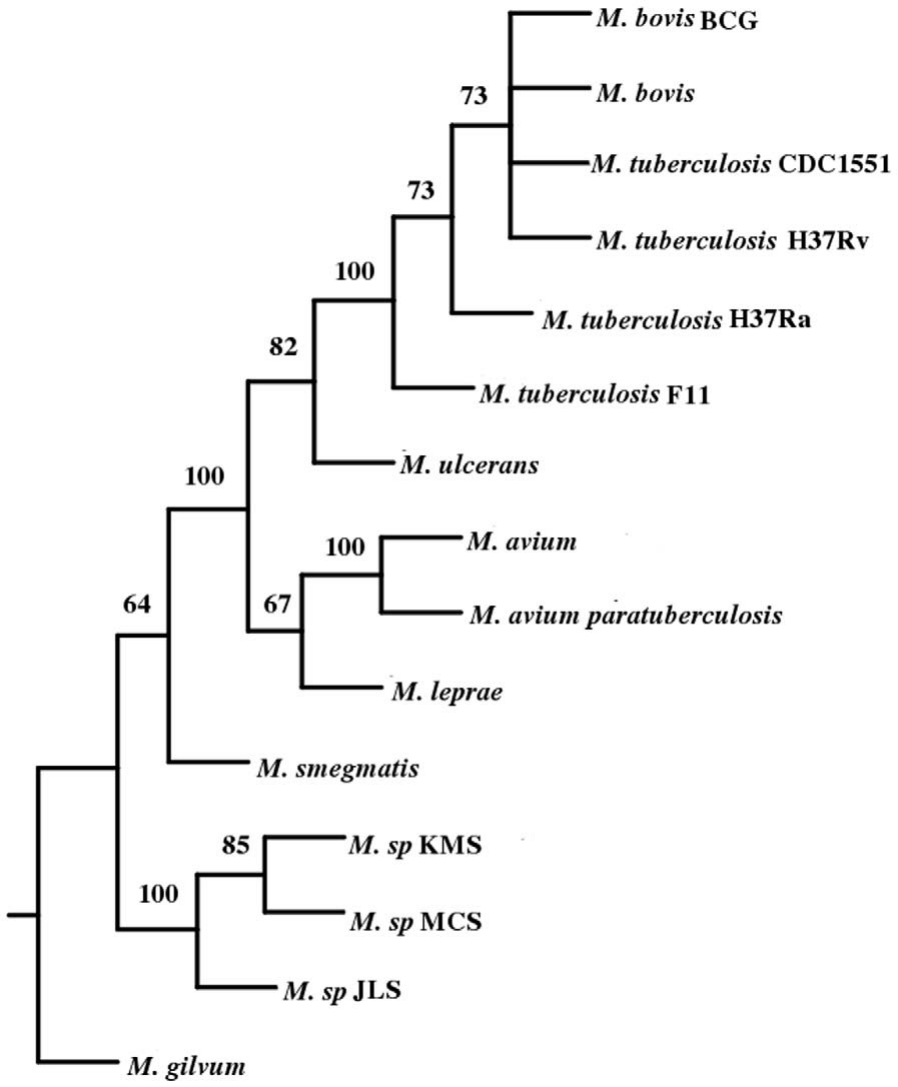
Phylogenetic tree of Mycobacterium based on 16S rRNA.

A phylogenetic tree was constructed based on pair wise distance computation of the fifteen fully sequenced strains and species of Mycobacteria (Fig. 2) using the Neighbor-Joining method of PHYLIP package [31], [32]. To validate the tree, bootstrapping was carried out as described in the “Methods”. The tree obtained was in agreement with the known relationship among the organisms. For example, organisms belonging to *M. tuberculosis* complex, such as different strains of *M. tuberculosis* and *M. bovis* are found in one cluster. There was a separation between fast growing *M. smegmatis*, *M. gilvum* and slow growing Mycobacteria as expected. Moreover, soil inhabitants *M. sp* MCS, *M. sp* JLS and *M. sp* KMS were also clustered separately from the slow growing Mycobacteria. The position of the members of tuberculosis complex with respect to that of *M. avium* sp paratuberculosis and *M. avium* 104 suggests that these are closer to the former than the fast growing Mycobacteria. This was also seen in the phylogenetic tree obtained using 16S rRNA [33]. In this study a clear separation of different strains of *M. tuberculosis* was observed. The strains H37Rv and Ra separated out from strains f11 and CDC1551. It was expected as the strain H37Ra is derived from Rv [34]. (Fig. 3). As expected, different strains were not resolved due to rRNA sequences being nearly identical in these strains.

**Figure 4:**
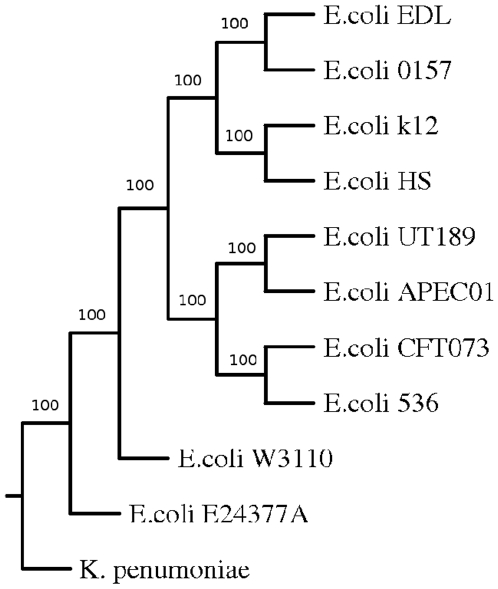
Phylogenetic tree of *E. coli* based on CNS.

Whole genome-based phylogenetic analysis was also carried out in order to check if this approach is able to capture biological relationships among another group of organisms. For this study different strains of enteric organism *E. coli* was used (Fig 4). The branches were found to be robust as most of the branches were supported by bootstrap values of 100%. The genomes of ten different strains of *E. coli* were clustered in two major groups. While non-pathogenic or pathogenic intestinal strains clustered together, uropathogenic strains were grouped separately. The two Enterohemorrhagic *E. coli* (EHEC) strains *E. coli* O157:H7 and E. coli EDL were in a different branches compared to Enterotoxigenic *E. coli* (ETEC) *E. coli* E24377A. The non-pathogenic laboratory strain *E. coli* K-12 and was found to be close to the commensal *E. coli* HS as expected. The uropathogenic strains *E. coli* UTI89,*E. coli* CFT073, *E. coli* 536 were grouped with the avian strain *E. coli* APECO1 into one cluster. All these strains cause extra intestinal disease and share the same set of virulence genes [35], [36]. Therefore the results presented here is consistent with the known biology of these organisms. We have also carried out analysis of different strains and species of Salmonella and found our results to reflect the phenotype of each individual strain or species (data not shown). Therefore it appears that CNS based distance estimate can capture evolutionary distance in a biologically meaningful way.

CNS is a simple distance measure based on a mismatch score. It does not account for the multiple substitutions present in the genomes and likely to miss some of the details about genome evolution. Therefore the Jukes-Cantor based distance was also calculated [37]. It corrects for multiple substitutions. The constant used in the distance calculation is 3/4 per nucleotide. There was no significance difference in the trees obtained using the CNS and Jukes-Cantor distance measures (data not shown here).We have also constructed phylogenetic tree using Maximum parsimony. The terminal branches on the tree obtained are not delineated as our method does (Fig S2). The reason is, Maximum parsimony is based on the mismatch scores of reconstructed ancestral sequences, which are similar in case of closely related sequences for example different strains of a species.

#### Indel-based tree

Sequence diversity is also due to insertions and deletions. Since these can also contribute to significant changes in phenotype of organisms, evolutionary distance can be determined using these events. As pointed out in “methods” the difference in the length of inter-anchor regions of S and the length between the corresponding anchors of T are due to either insertion/deletion or expansion/contraction of repeats. This difference was used to calculate pair wise distance between the two genomes using two different approaches. In the first approach the distance was based on the number of nucleotides that vary between the homologous anchors (Inter-Anchor Distance 1 - IAD 1) whereas Hamming distance based on binary events was used as the second distance measure ( Inter-Anchor Distance 2 - IAD 2). In IAD 1 length of the indel determines the score. Difference in every nucleotide is considered as an independent event. On the contrary IAD 2 assumes indels as single event irrespective of the size and gives equal weights to all the events. For this study we have taken only the conserved anchors present in the genomes.

**Figure 5:**
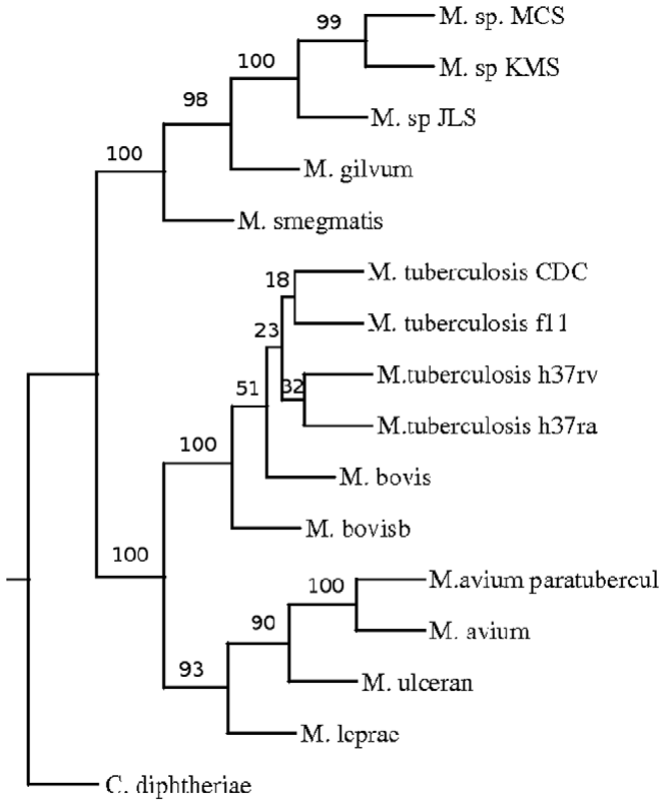
Phylogenetic tree of Mycobacterial genomes based on inter anchor Distance (IAD 1).

**Figure 6:**
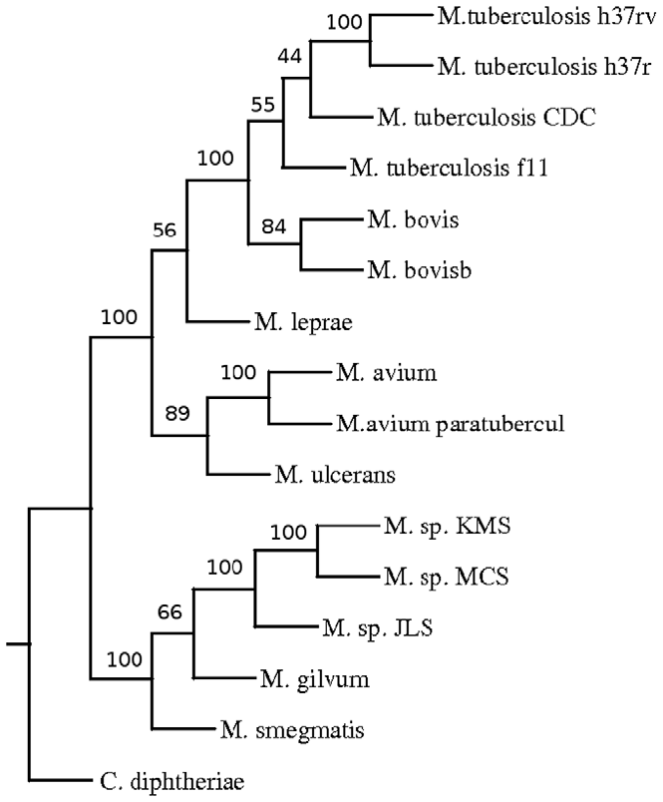
Phylogenetic tree of Mycobacterial genomes based on inter anchor distance (IAD 2).

In general the phylogenetic trees obtained by these approaches were found to be quite similar to that obtained by using CNS (Fig. 2,5,6). Interestingly when IDA 2 was used for the analysis, all the *M. tuberculosis* isolates clustered together suggesting that the number of indels may be very similar in these organisms. The positions of *M. leprae*, *M. ulcerans* were different compared to the tree derived by using IAD 1 (Fig 6). Some of these organisms have undergone deletions during evolution, for example *M. ulcerans* has lost 102 genes compared to that of *M. tuberculosis* [38] and *M. leprae* has undergone large scale secondary loss of genes [39].

**Figure 7:**
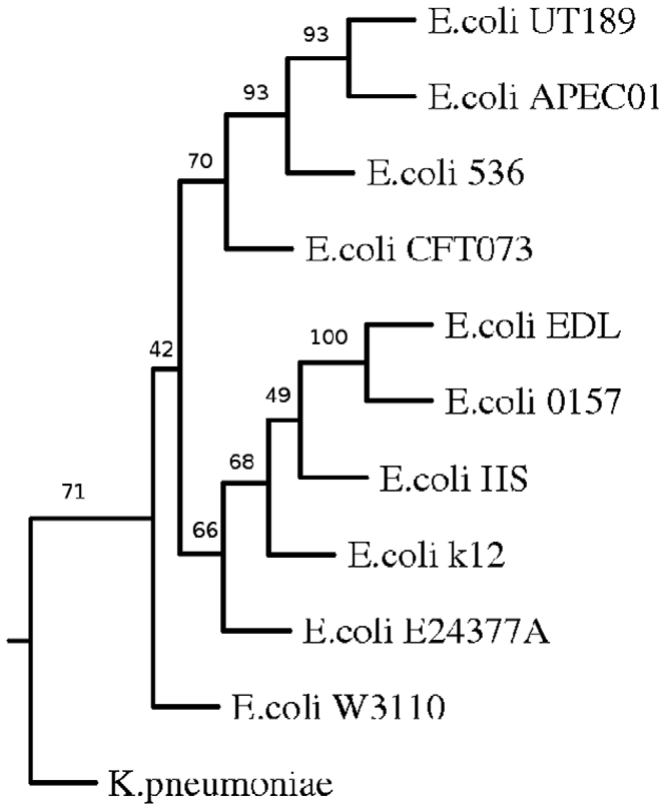
Phylogenetic tree of E. coli genomes based on inter anchor distance. Phylogenetic tree of different strains and species of *E. coli* based on IAD 1.

**Figure 8:**
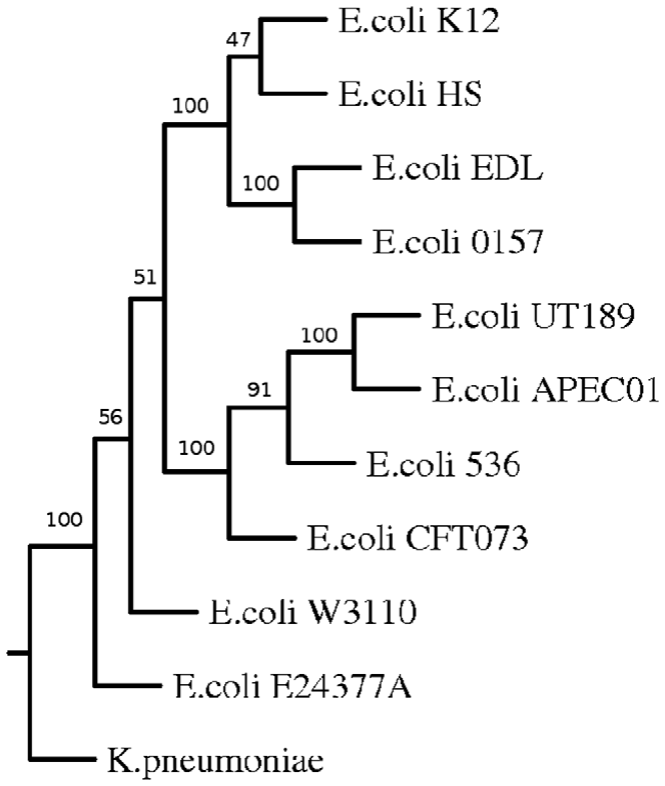
Phylogenetic tree of E. coli genomes based on inter anchor distance. Phylogenetic tree of different strains and species of *E. coli* based on IAD 2.

The trees obtained by using indels as a measure were found to be similar to that obtained using CNS except the position of *E. coli* CFT073. The *E. coli* genome is a mosaic with the backbone of genes disrupted by insertions of genomic regions by horizontal gene transfer. It is likely that the patterns of horizontal gene transfer events in uropathogenic strains were different and that small indels may have played a more important role in their evolution [40] ( Fig. 7,8).

#### Anchor order-based trees

**Figure 9:**
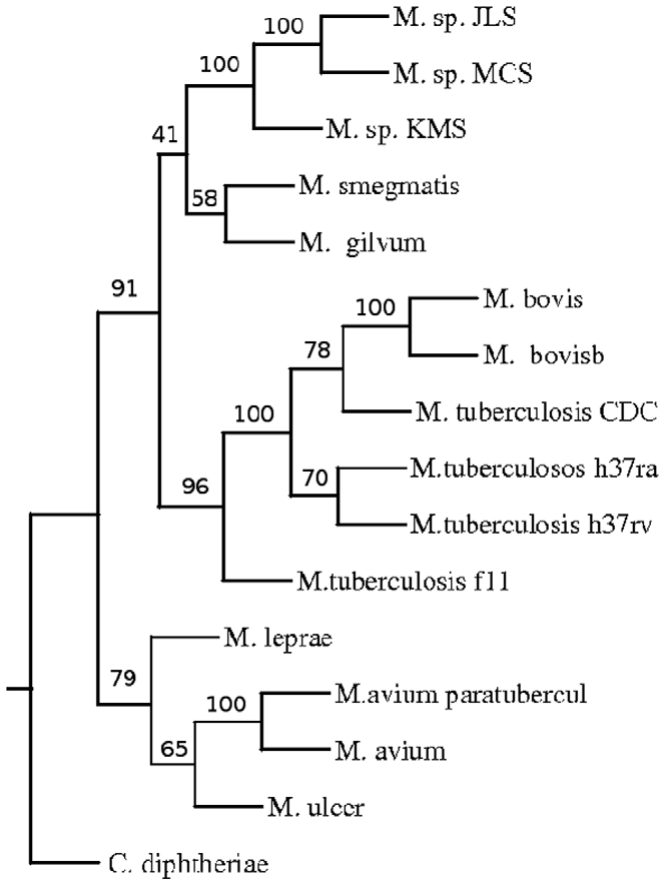
Phylogenetic tree of *M. tuberculosis* genomes based on Anchor order.

The changes in gene order has also been used to determine phylogenetic distance among organisms [41], [42]. The evolutionary mechanisms, such as recombination, shuffle the order of the genes leading to disruption of syntenic relationship. The degree of conservation of synteny can therefore, be used for deciphering evolutionary relationships. We have used the degree of conservation of anchor order to calculate pair wise distance among genomes. A small set of anchors (400) were found to be conserved across all the species of Mycobacteria and these were used for the analysis (Fig 9).

In the resultant tree the position of *M. tuberculosis* CDC1551 was different compared to the tree derived using CNS among *M. tuberculosis* strains. This may be due to comparatively smaller number of insertion elements in *M. tuberculosis* CDC1551 and consequently lower rate of recombination. It is known that IS elements are likely to be preferred sites of recombination due to high sequence identity [43].

**Figure 10:**
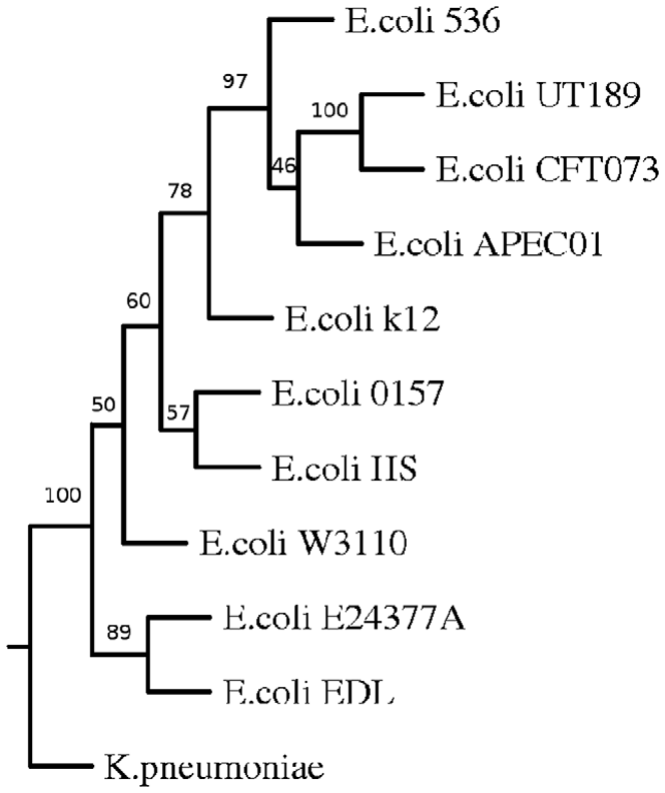
Phylogenetic tree of *E. coli* genomes based on Anchor order.

Genome rearrangement leading to changes in anchor order may be the major factor for the placement of *E. coli* CFT073 [44] (Fig. 10). The other uropathogenic strains have common branch point signifying that the anchors in these three organisms have maintained synteny. However, enterohemorrhagic strains *E. coli* (EHEC), *E. coli* O157:H7 was clustered with commensal *E. coli* HS. This suggests that the genome rearrangements took place before enterohemorrhagic and non pathogenic *E. coli* separated out [45].

### Construction of Tree from Supermatrix

**Figure 11:**
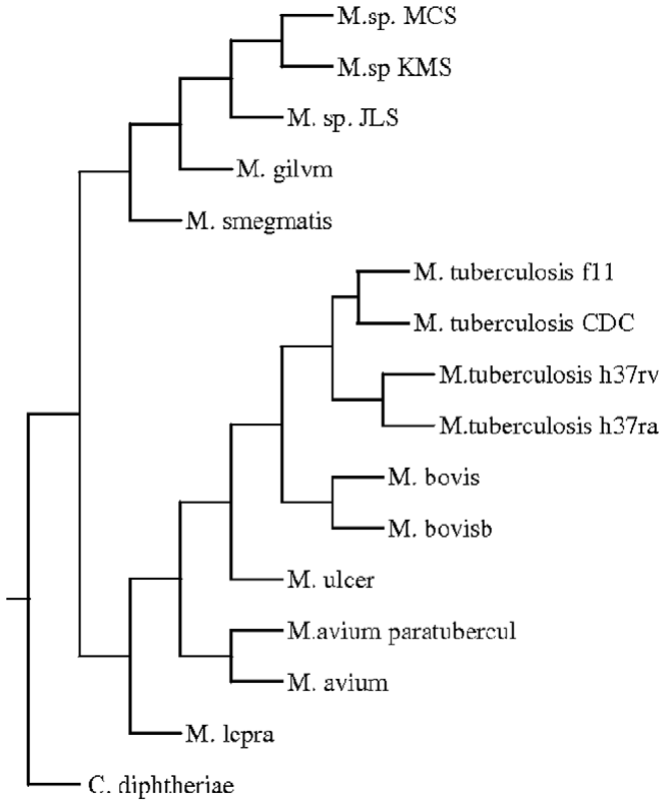
Super Phylogenetic tree of Mycobacteria genomes.

The trees constructed by different distance measures revealed the role of different molecular events in the evolution of the genomes of the organisms under study. The comparison of different trees showed that there are differences between them in the positioning of some of the strains and species, for example the position of *M. tuberculosis* CDC1551 is similar in trees obtained from CNS, IAD 2 and anchor order but different in the tree constructed using IAD 1. In order to get a true evolutionary relationship it is important to derive a single tree based on multiple distance estimates encompassing different molecular events. To fulfill this aim we constructed a tree which is based on a pair wise distance that is an average of all the different distance measures described here. The resultant tree is shown in Figure 11. The relationships observed, correlates with the pathogenic importance of different Mycobacteria centered on their ability to infect and cause disease among mammalians (humans, domesticated animals and wild life). A clear separation of non-pathogenic, saprophytic Mycobacteria, such as *M. smegmatis*, *M. gilvum* and others, separate out as a cluster from the rest of the inherent pathogenic mycobacterial species. Further this criterion of the phylogenetic relationship confirms the pathogenic hierarchies seen among the known Mycobacterial pathogens of humans and animals. *M. avium* is known to infect cattle and is associated with infection among immuno-compromised humans, such as HIV infected and transplant patients undergoing immunosuppressive therapy [46]. More potent disease producing mycobacteria branch out next, namely *M. avium* subsp paratuberculosis, *M. ulcerans* and *M. leprae*. *M. avium* subsp paratuberculosis is associated with Crohns disease in humans and Johnes disease in sheep [47]. *M. ulcerans* and *M. leprae* are associated with human skin / dermal infection. *M. leprae* is distinct from *M.ulcerans* and is more closely related to members of the *M. tuberculosis* complex. However the distinction between *M. leprae* and tuberculosis complex is evident by the analysis. Further the tuberculosis complex is separated into *M. tuberculosis* and *M. bovis*. These two species are notoriously identical at the genome level. By this unique classification they branch out distinctly from *M. tuberculosis*. The separation of these two pathogenic species capable of being the cause of a common human and bovine disease, namely tuberculosis, reflects the usefulness of the outlined phylogenetic tree. These two mycobacteria cause disease across species namely Zoonotic / reverse zoonotic tuberculosis.

The composite tree of *E. coli* was found to be nearly identical to that obtained using CNS (data not shown).

## Discussion

Evolutionary relationships have been traditionally deciphered using sequences derived from rRNA and occasionally a few conserved proteins. These approaches are not suitable to analyze terminal branches and very closely related organisms. This is evident from the fact that the nucleotide sequence of 16S rRNA of the two strains of *M. tuberculosis*, *M. tuberculosis* CDC1551 and *M. tuberculosis* H37Rv is identical. Moreover, rRNA sequences are only a small fraction of any genome and therefore do not reflect changes that occur at the whole genome level. Whole genome sequences provide detailed information about an organism and evolutionary relationship derived from these may be more accurate. Availability of whole genome sequences of a large number of organisms does provide enough data to derive biologically meaningful relationships and understand the basis of phenotypic divergence. Genomes not only evolve at the level of nucleotide sequence, but also overall organization that include indels and rearrangement leading to sequence reorganization. Therefore, evolutionary distance should involve in principle all the different features.

In this study a number of different approaches have been used to derive distance measures for constructing phylogenetic trees. All the approaches use complete genome sequences and the different measures described here reflect different mechanism by which genomes evolve. For example, SNPs mainly contribute to CNS. Some of the distance measures used in this study are derived from insertion/deletion and recombination, processes by which organisms diverge from each other. So far there has not been a single study where all these different mechanisms-based distance estimates have been used for deciphering phylogenetic relationships though some of the mechanisms have been tried individually, for example, trees have been derived based on maintenance of gene synteny [42].

In the approach described here random identification of anchors has been used for sampling different regions of the genome without any bias. Since 10% of the genome is sampled the results would statistically give an overall picture of the genomes [30]. Moreover, due to random selection of anchors the effects of base compositional bias, horizontal gene transfer and different rates of evolution at different locations would be negligible. It was also shown by Rokas *et al* [29] that 8000 randomly selected nucleotides, is enough for producing the correct phylogeny. Similar result was also obtained in this study. The number of chosen anchors was found to be more than sufficient for obtaining a unique and robust value of CNS that is independent of sampling error. The results were also found to fit the correct understanding about the biology of the organisms. Overall all the different trees drawn using distance measures derived from CNS, anchor length variation and changes in anchor order were found to be similar maintaining the position of many of the branches and clusters of organisms with some minor exceptions. For example, the trees derived by using CNS placed *M. leprae* and *M. avium* together in one branch. However, these were placed in different positions in the trees computed using measures derived from changes in inter-anchor length. Due to genome decay and gene loss *M. leprae* is much shorter than other Mycobacterium [39], [48]. This led to a major change in inter anchor lengths and consequently a different position in the tree. The position of *M. ulcerans* also showed variation in different trees and this can also be attributed to large scale horizontal gene transfer and reductive evolution leading to genomic rearrangements and deletions [38]. One of the major advantages of the method described here is its ability to analyse closely related organisms, such as different strains of the same species. Our attempt to generate a composite tree which would reflect genomic changes brought about by different molecular mechanisms was very encouraging as the derived tree was able to explain biological and clinical relationships among the organisms.

A number of studies have been carried out to identify diverse regions in number of isolates of *M. tuberculosis* complex utilizing a variety of experimental approaches, such as genomic microarray, PCR amplification and restriction polymorphism [49]–[51]. The results suggest that the evolution of different strains and species is aided by frequent insertion/deletions, duplication and recombination processes rather than sequence divergence [34], [43], [52]. Particularly insertion elements have played a significant role in these processes [43], [53]. Attempts to derive phylogenetic relationships have not been very successful as different markers lead to different results and none of the markers can correctly capture the variations as these are caused by multiple mechanisms. For example, *M. tuberculosis* CDC1551 was found to be closer to *M. bovis* compared to *M. tuberculosis* H37Rv when membrane lipoprotein was used as a marker [43]. On the other hand a different result was obtained when the tree was constructed using adenylate cyclase sequences [43]. Since most of the studies involved in comparing different strains and species take into account data from a few markers it is likely that the results may not reflect true relationship. Our data clearly show that *M. tuberculosis* H37Rv may have undergone more genomic changes as compared to *M. tuberculosis* CDC1551. This may be due to the fact that the strains H37Rv and Ra are in culture for a long time and other strains have been recently isolated. All the organisms belonging to *M. tuberculosis* complex may have evolved from a common ancestor. This is also inferred from some of the sequencing experiments of a large number of field isolates [54].

The CNS based *E. coli* tree was able to capture the phenotypic differences due to adaptation to specific ecological niche. For example, uropathogens were well separated from the intestinal pathogens and non-pathogens. Therefore, CNS turns out to be a good parameter for estimating the relationship among the organisms as the core features were captured and was not affected by horizontal gene transfers. Since *E. coli* genome has a number of horizontally transferred genes many methods that compute phylogenetic trees do not give correct relationship [55]. The non-pathogenic *E. coli* can become a pathogen simply by acquisition of toxin genes as suggested by Turner *et al* [56]. It was also shown that ETEC strain ( *E. coli* H10407) is 96% similar to the non-pathogenic *E. coli* K12 MG1655 and the differences are mainly due to the genes which cause virulence [57]. In our study in tree based on insertion and deletions also grouped ETEC strain *E. coli* E243 with non-pathogenic strain *E. coli* K12 suggesting that the method described here is capable of deciphering biological relationship.

In conclusion our results show that random anchor based approach with multiple distance measures can be very useful in comparative genomics, particularly in deciphering evolutionary relationships among organisms and identifying diverse regions in different genomes. In the studies shown here our approach has often be able to explain the underlying biological phenomenon not approachable by other methods.

## Methods

### Selection of anchors and finding homologous anchors

Let S (the query) and T (the target) be two genomes of lengths N and M respectively. We first select some random positions on the query genome. Each of these positions would be starting points of the anchors. The anchors are of fixed length m and we require that these anchors be non-overlapping. As such we need to ensure that there is a minimum distance, $L$, between two successive random positions, where L> = m. We obtain this as follows.

Let x _1_, x _2_, … , x _N_ be a random permutation of the numbers 1,2,… , N, where each permutation is equally likely to occur. This random permutation is obtained by the Mersenne Twister programme (http://www.math.sci.hiroshima-u.ac.jp/~m-mat/MT/emt.html). The random positions of the anchors are constructed according to the following iterative scheme, let y _1_ = x _1_; and y _2_ = x _k 1_, where k _1_ = j>1, |x _j_−y _1_ |> = L; having defined y _i_ and k _i−1_ let y _i+1_ = x _ki_ where k _i_ = min j>k _i−1_,|x _j_−y_l_|> = L for all l< = i.

We terminate this iterative scheme when it is not possible to define any further y. Let y _1_ ,y _2_ ,…,y _n_ be the set of all possible y's obtained by the above scheme.

We note here that y _1_ ,y _2_ ,…,y _n_ need not be in either an increasing or a decreasing order. However, with a slight abuse of notation assume that y _1_ ,y _2_ ,…,y _n_ are in an increasing order.

Let λ _i j_ denote the nucleotide at the position j+y _i_ in the query genome S. Thus, for example λ _i j_ = A if the nucleotide at the (j+y _i_ )th position in the query genome S is A, etc.

The string
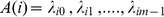
(1)represents the string consisting of m consecutive nucleotides of the genome S starting at the y _i_ th position.

The strings A(1), A(2), … , A(n) represent our anchors at positions y _1_ ,y _2_ ,…,y _n_ on the genome S. The choice of y _i_ 's ensure that these anchors do not overlap.

Based on these anchors we obtain a set of strings B(1), B(2), … , B(n) from the target genome T. The string B(i) is that segment of T which gives the highest BLAST score when compared with the string A(i) of the query genome S.

To fix notation let the string B(i) start from the position t _i_ of the target genome T. Letting μ_ij_ denote the nucleotide at the positiont_i+j_ in the target genome, we have
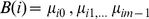
(2)We note that B(i)'s may be overlapping, and although A(i)'s are arranged in an increasing order according to their position in the genome S, B(i)'s need not preserve that order.

Let
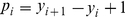
(3)

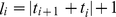
(4)A distance based on mismatches
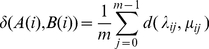
(5)where
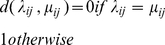
(6)The mismatch score is
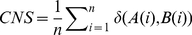
(7)Since this distance between S and T is not reflexive, in the sense that d(S,T) need not equal d(T,S), we enforce it to be so by symmetrizing and defining the following distance
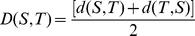
(9)Nonetheless, D(., .) is not a distance metric – the triangular inequality may not be satisfied. To see this consider the following pathological example.

Let a_1_,a_2_,…, b_1_,b_2_,…,c_1_,c_2_,…,d_1_,d_2_.…, and e_1_,e_2_ …. be strings of nucleotides each of length m and consider the following three ‘artificial’ genomes S = a_1_, b_1_, c_1_, a_2_, b_2_, c_2_, … T = b_1_, d_1_, a_1_, b_2_, d_2_, a_2_,… R = d_1_, c_1_, e_1_, c_2_, b_2_, e_2_,… For a_1_,a_2_,… as a random position in genome S. d(S,T) = 0 whereas d(S,R) = 1 Similarly for genome T if b_1_,b_2_,… are the random positions d(T,S) = 0 whereas d(T,R) = 0 For genome R if d_1_,d_2_.… are taken as random samples. d(R,S) = 1 whereas d(R,T) = 0.

Thus D(S,T)+D(T,R)> = D(S,R) does not hold. This example is indeed a ‘pathological’ one as described earlier, because in practice, as may be seen from table Table 1 with real-life genomes and most random positions, the triangular inequality is indeed valid.

**Table 1:** Pairwise distances of different set of genomes.

S Genome/T Genome	*M. tuberculosis* CDC1551	*M.tuberculosis* H37Rv	*M.bovis*
*M. tuberculosis* CDC1551	0.0000	0.0094	0.0184
*M.tuberculosis* H37Rv	0.0103	0.0000	0.0188
*M.bovis*	0.0096	0.0105	0.0000

### A distance based on inter-anchor regions IAD 1

We construct a distance measure based on the inter-anchor separation distance as follows and p _i_ and l _i_ are described earlier in equation (3) and (4):

For i = 1, …, n−1, where p _i_ and l _i_ are described earlier.
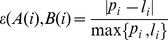
(10)and
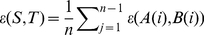
(11)Again to ensure reflexivity, we symmetrize it by taking as our distance
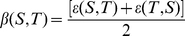
(12)


### A distance based on Hamming Distance IAD 2

The events which occur at gross level in the genome like indels, rearrangements, translocation, inversion all are given equal weightage. The inter-anchor length difference of anchors in genome S and genome T which are greater than 2 are taken for study and the Hamming distance is defined as:

For i = 1, … , n−1,
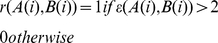
(13)

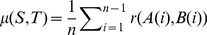
(14)and
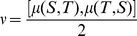
(15)


### A distance based on anchor order

The gene order approach used depends on the conservation of the genes, we construct a distance measure based on the same approach taking the anchor order as follows:

For i = 1, 2, …, n−2 let o(A(i), B(i)) be given by
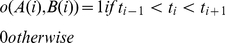
(16)

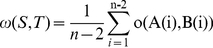
(17)

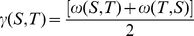
(18)


### Bootstrapping

The distance between the genomes S and T is calculated using the scores of n anchors. To estimate the confidence in the constructed phylogenetic tree using CNS, we carried out the bootstrapping. In this procedure, the resampling of the scores of n anchors with replacement is carried out for CNS calculation. This is repeated 1000 times. Therefore, 1000 trees are generated and a consensus tree is obtained by majority rule. The bootstrap value obtained for each node is the number of times that nodes appeared in all the 1000 trees generated, thus is the measure of confidence of the occurrence of the node in the phylogenetic tree.

### Phylogenetic tree construction

The distance measure obtained by all the methods described is used to get all the pairwise distance between Mycobacterial genome and Streptococci. The distance matrix obtained for all the genomes is used to construct the phylogenetic tree using the Neighbor Joining [31] method of PHYLIP package [32].

### Data

The genomes of Mycobacteria which were analyzed *M. tuberculosis* CDC1551, *M. tuberculosis* H37Rv, *M. tuberculosis* H37Ra, *M. bovis*, M. bovis BCG str. Pasteur 1173P2, *M. avium* and *M.leprae M. tuberculosis* F11, *M sp*. KMS, *M sp*. MCS, *M. sp*. JLS, *M. avium* subsp. paratuberculosis K-10, *M. gilvum* PYR-GCK and *M. ulcerans* Agy99 were obtained from NCBI (http://www.ncbi.nlm.nih.gov/genomes/lproks.cgi), *M.smegmatis* was obtained from (ftp://ftp.tigr.org/), *M. marinum* was obtained from (ftp://ftp.sanger.ac.uk/pub/pathogens/mm/MM.dbs). The genomes of all strains of *E.coli* such as *E. coli* 536 , *E. coli* APEC01, *E. coli* CFT073, *E. coli* E24377A, *E. coli* HS , *E. coli* K12 , *E. coli* O157:H7 EDL933, *E. coli* O157:H7 str Sakai, *E. coli* UT189 , *E. coli* W3110, and *S. enterica* subsp enterica serovar Paratyphi A str ATCC 9150 were obtained from NCBI.

## Supporting Information

Figure S1 Schematic flow diagram of Methodology.
(0.38 MB EPS)

Figure S2 Phylogenetic tree of *M. tuberculosis* genomes based on Maximum Parsimony.
(1.61 MB TIF)
