# Correction: Anchor-Based Whole Genome Phylogeny (ABWGP): A Tool for Inferring Evolutionary Relationship among Closely Related Microorganisms

**DOI:** 10.1371/annotation/a56d6c19-b938-4733-8333-79ff48202414

**Published:** 2010-12-30

**Authors:** Anchal Vishnoi, Rahul Roy, Hanumanthappa K. Prasad, Alok Bhattacharya

The word "microorganism" is misspelled in the article title. The correct title is "Anchor-Based Whole Genome Phylogeny (ABWGP): A Tool for Inferring Evolutionary Relationship among Closely Related Microorganisms" The correct citation is Vishnoi A, Roy R, Prasad HK, Bhattacharya A (2010) Anchor-based whole genome phylogeny (ABWGP): A tool for inferring evolutionary relationship among closely related microorganisms. PLoS ONE 5(11): e14159. doi:10.1371/journal.pone.0014159

