# Supplementary figures and images for: Anchor-Based Whole Genome Phylogeny (ABWGP): A Tool for Inferring Evolutionary Relationship among Closely Related Microorganims
Source: PLoS One. 2010 Nov 30;5(11):e14159. doi: 10.1371/journal.pone.0014159 (PMC2994773; doi:10.1371/journal.pone.0014159)

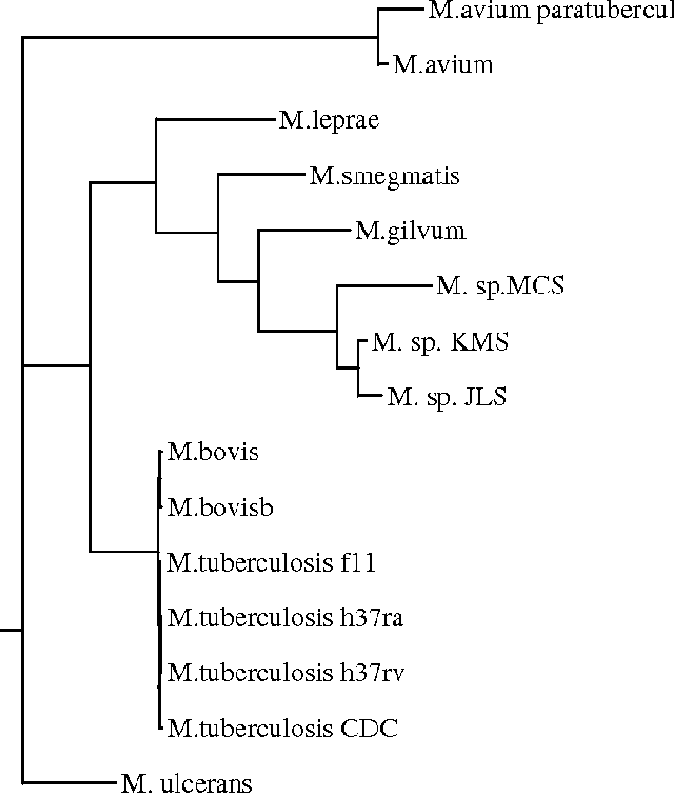

Supplement: Figure S2 — Phylogenetic tree of M. tuberculosis genomes based on Maximum Parsimony. (1.61 MB TIF) [file pone.0014159.s002.tif]
